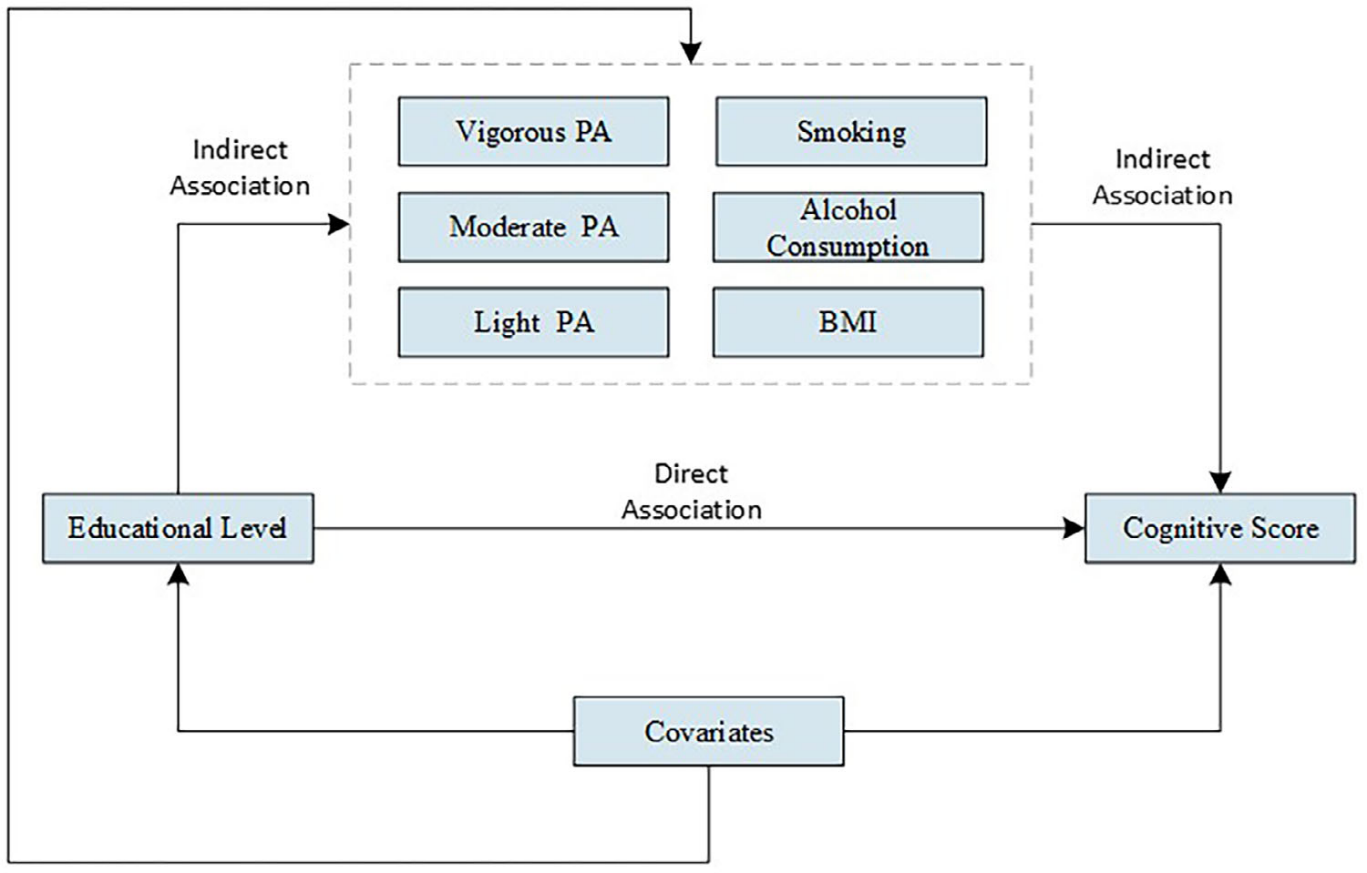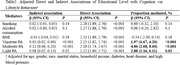# Educational Level and Cognition: The Mediating Effects of Lifestyle Behaviors

**DOI:** 10.1002/alz70860_101709

**Published:** 2025-12-23

**Authors:** Yufei Wang, Lillian Morgado, Catherine E. A. Scipion, Jalayne J. Arias

**Affiliations:** ^1^ Georgia State University, Atlanta, GA, USA; ^2^ Centers for Disease Control, Atlanta, GA, USA; ^3^ School of Public Health ‐ Georgia State University, Atlanta, GA, USA

## Abstract

**Background:**

Evidence indicates education is a protective factor on cognitive function. The mechanisms through which education is protective are unknown. Prior research has indicated that lifestyle may play a factor in mitigation role education has in protecting against the risk of Alzheimer's Disease and cognitive impairment. Yet, evidence is scarce. This study aims to explore the potential mediating role of lifestyle factors on the education‐cognition relationship.

**Method:**

Data were derived from Waves 8–13 of the Health and Retirement Study, including 1,752 Americans aged ≥50. Based on Wave 8 data, participants' education levels were categorized as “High school or less” or “College degree or above.” Cognitive function at Wave 13 was assessed using the Telephone Interview of Cognitive Status. Ten years of lifestyle behavior data, including smoking, alcohol consumption, BMI, and physical activity, were collected from Waves 8–13. We used mediation analyses to explore the direct and indirect effects of education on cognitive function in R Version 4.3.1.

**Result:**

Moderate physical activity had the highest mediation effect [4.86%, 95%CI:(2.68%, 8.60%)], followed by light physical activity [2.88%, 95%CI: (1.16%, 6.11%)] and vigorous physical activity [1.97%, 95%CI: (0.67%, 4.20%). Other lifestyle variables had no significant mediation effect on the relationship between education and cognitive function.

**Conclusion:**

Physical activity potentially mediates the education‐cognition relationship. In cognitive function research or intervention projects, the lifestyle of people having low education levels warrants further attention.